# Manufacturing of Human Extracellular Vesicle-Based Therapeutics for Clinical Use

**DOI:** 10.3390/ijms18061190

**Published:** 2017-06-03

**Authors:** Mario Gimona, Karin Pachler, Sandra Laner-Plamberger, Katharina Schallmoser, Eva Rohde

**Affiliations:** 1 GMP Unit, Spinal Cord Injury and Tissue Regeneration Center Salzburg (SCI-TReCS), Paracelsus Medical University (PMU), 5020 Salzburg, Austria; mario.gimona@pmu.ac.at (M.G.); karin.pachler@pmu.ac.at (K.P.); s.laner-plamberger@salk.at (S.L.-P.); k.schallmoser@salk.at (K.S.); 2 Research Program Nanovesicular Therapies, Paracelsus Medical University (PMU), 5020 Salzburg, Austria; 3 Department of Blood Group Serology and Transfusion Medicine, Paracelsus Medical University (PMU), 5020 Salzburg, Austria

**Keywords:** extracellular vesicles, exosomes, vesicular secretome fraction, mesenchymal stromal cells, therapeutics, critical size bone defect, epidermolysis bullosa, spinal cord injury, good manufacturing practice

## Abstract

Extracellular vesicles (EVs) derived from stem and progenitor cells may have therapeutic effects comparable to their parental cells and are considered promising agents for the treatment of a variety of diseases. To this end, strategies must be designed to successfully translate EV research and to develop safe and efficacious therapies, whilst taking into account the applicable regulations. Here, we discuss the requirements for manufacturing, safety, and efficacy testing of EVs along their path from the laboratory to the patient. Development of EV-therapeutics is influenced by the source cell types and the target diseases. In this article, we express our view based on our experience in manufacturing biological therapeutics for routine use or clinical testing, and focus on strategies for advancing mesenchymal stromal cell (MSC)-derived EV-based therapies. We also discuss the rationale for testing MSC-EVs in selected diseases with an unmet clinical need such as critical size bone defects, epidermolysis bullosa and spinal cord injury. While the scientific community, pharmaceutical companies and clinicians are at the point of entering into clinical trials for testing the therapeutic potential of various EV-based products, the identification of the mode of action underlying the suggested potency in each therapeutic approach remains a major challenge to the translational path.

## 1. Introduction

Released membrane vesicles from pro- and eukaryotic cells, such as exosomes, microparticles, microvesicles and apoptotic bodies, represent a dynamic extracellular vesicular compartment that is increasingly recognized in basic research and translational clinical development [1]. Currently, the biophysical and immunochemical characterization of extracellular vesicles (EVs) and their essential paracrine or autocrine biological effects are areas of intense investigation due to their anticipated diagnostic or therapeutic value. In the late 1990s, the concept of secreted EVs as an alternative to cellular therapies emerged, supported by data indicating that established murine tumors could be eradicated by dendritic cell (DC)-derived exosomes [2]. This seminal study was soon followed by a first report on the clinical grade production and characterization of DC-exosomes in 2002 [3]. In addition, two early phase I clinical trials demonstrated feasibility, safety and low toxicity of exosomes derived from tumor peptide-loaded DCs administered to patients suffering from metastatic melanoma or non-small cell lung cancer [4,5]. However, a recent phase II trial testing exosomes from interferon gamma-matured DCs loaded with MHC class I- and class II-restricted cancer antigens failed to reach the primary endpoint of at least 50% patients with progression-free survival at 4 months after chemotherapy cessation [6].

Although the molecular basis for the therapeutic effect of the reported DC-exosome preparations in cancer patients remains unclear, these pioneering studies are important, as they provoked great scientific interest regarding the therapeutic potential of EVs or vesicular secretome fractions (VSFs). An extended overview of non-clinical and clinical studies investigating EVs as novel therapeutic agents in anti-tumor immune therapy, against infectious diseases, in immunomodulatory and regenerative therapies, and as drug delivery systems was compiled recently in a position paper by the International Society for Extracellular Vesicles (ISEV) [7]. Suggestions for the pharmaceutical categorization of novel EV-based therapeutics, as well as recommendations for structured procedures according to pharmaceutical quality requirements, have been delineated by experts from the ISEV together with participants of a Cooperation in Science and Technology (COST) action (European Network on Microvesicles and Exosomes in Health and Disease, Me-HaD), supported by the EU Framework Program Horizon 2020. Although the number of clinical trials registered in international databases has increased from 29 to 52 within one year, only two of the listed studies investigate exosomes as therapeutics (search term “exosome” and search dates 29 June 2016 and 13 April 2017 at www.clinicaltrials.gov). Searching for the terms “extracellular vesicles or EVs and therapeutics” at www.clinicaltrials.gov and at www.clinicaltrialsregister.eu did not uncover additional registered studies, confirming that clinical translation is still in its infancy.

Nevertheless, several companies have entered the stage in the meantime, and offer various EV-based therapies for a range of disease conditions and therapeutic targets (listed in Table 1). Robustness and reproducibility of many of these proposed approaches, however, await support by solid preclinical and early clinical data [8]. While few companies have well developed and focused portfolios (e.g., ReNeuron, Capricor, Codiak), others have just passed the phase of establishment. However, the increasingly competitive nature of translation and business-oriented activities indicates that many independent academic and industrial research groups have identified biologically relevant effects of EVs or VSFs that can be exploited for a number of novel therapies.

Despite the many open questions in the field, we witness a strong movement towards the development of EV-based therapies. It can be envisaged that EVs or VSFs of unmanipulated or engineered human cells represent the therapeutically active substance of these biologic drugs [9,10,11,12,13,14,15,16,17,18,19,20,21,22,23]. To make these therapeutics available for patients in the future, it is necessary to develop scalable, reproducible and good manufacturing practice (GMP)-compliant manufacturing protocols while recognizing the benefit of regulatory frameworks.

## 2. Background

### 2.1. Therapies Using MSC-EVs and not Necessarily MSCs

Clinical testing of investigational medicinal products is generally underpinned by preclinical test programs that span from discovery phase and proof-of-concept (PoC) studies to definitive safety trials. The design, conduct and interpretation of results from the preclinical phase is critical to justify further testing in humans [24]. Development of cell- or EV-based therapeutics is decelerated by road blocks like inherent heterogeneity and biological or technological complexity, which hamper the finding of the therapeutically active substance (i.e., active component) and its definite mode of action (MoA). The MoA of EV-based therapeutics derived from in vitro expanded cells can be influenced not only by the parental cell type, but also by modifications in handling, culture conditions, and materials or medical devices used for EV administration.

For advancing the development, it is helpful to define early on the manufacturing steps and characterization procedures for EV-based therapeutics. The same applies to in vitro and in vivo potency assays, which have to be established to systematically evaluate the expected biological activity/ies (or therapeutic potency/ies) in adequate models. It is also crucial for the design of preclinical testing programs to focus on target disease(s) at early stages of drug development, and to consider appropriate, biologically relevant animal models. Specifically, preclinical testing programs will help (1) to establish the rationale for a proposed therapeutic approach; (2) to identify, characterize, quantify and minimize toxicities and uncover dose-toxicity relations; (3) to select safe initial clinical starting doses, dose escalation schemes and dosing regimens; and (4) to define subject eligibility and clinical monitoring strategies [24].

For the initiation of early clinical trials, a defined MoA is not mandatory, but a plausible hypothesis about a suggested MoA will be requested by health authorities [7]. Another decisive element is the identification of the “active drug substance(s)”; that is, the component(s) responsible for one or more purported MoA/s responsible for the biological activity/ies of novel EV-based therapeutics. Owing to the many variables that must be considered for each intended therapeutic approach, we restrict the discussions in this article to possible strategies for developing EVs or VSFs derived from a particular and unmodified human cell type, the mesenchymal stromal cell (MSC). We highlight the scientific rationale for the proposed MSC-EV therapies and their probable paths from laboratory to clinical use, focusing on selected target diseases with a clear unmet medical need—(1) critical size bone defects, (2) epidermolysis bullosa (EB) and (3) spinal cord injury (SCI)—to illustrate a necessary and manageable case-by-case approach to the design of preclinical testing programs, which are often executed for biological drug development.

### 2.2. Critical Size Bone Defects

Mesenchymal stromal cells have been applied allogeneically in the clinic to correct the genetic disorder osteogenesis imperfecta [25], and have been used for autologous therapy in combination with platelet-rich plasma and/or scaffolds for distraction osteogenesis to treat limb length discrepancy or large bone defects in a number of studies [26,27,28,29]. Recent animal data confirm that the combination of MSCs with platelet-rich plasma most efficiently promoted bone regeneration in the presence of high calcium levels [30]. In addition to these direct, pro-regenerative and tissue reconstructive approaches, immunomodulatory intervention strategies have been reported to maximize regenerative and minimize destructive effects of inflammation leading to enhanced bone fracture healing in animal experiments [31,32,33]. Considering the immunomodulatory potential of MSC-EVs (see Pachler et al., submitted to this issue, and [34,35,36]), future clinical studies may not only test MSCs, but also MSC-EVs for their capacity to modulate inflammation in the context of bone regeneration. An exosome-mediated mode of communication between mineralizing osteoblasts and stromal cells in the bone microenvironment was shown to induce osteogenic differentiation in vitro [37]. Promising results on the pro-osteogenic impact of MSC-EVs have been obtained from rat models: MSC-EVs were shown to stimulate bone regeneration in a critical size calvarial bone defect model [38], to promote cartilage restoration and subchondral bone regeneration in a critical-sized osteochondral defect [39], and to prevent bone loss and enhance angiogenesis in a femoral head osteonecrosis model [40]. Further evidence for a pro-bone regeneration capacity along with a pro-angiogenic capacity of EV-modified scaffolds in a mouse model [41] emphasizes that EV-enriched scaffolds represent a possible therapeutic solution for the unmet need of healing critical size bone defects.

### 2.3. Epidermolysis Bullosa (EB)

The skin fragility disorder recessive dystrophic epidermolysis bullosa (RDEB) belongs to a group of rare genetic skin diseases characterized by detachment of skin layers due to reduced or lacking type 7 collagen (C7) or defective anchoring fibrils at the dermal-epidermal junction [42]. Currently there is no cure for the disorder and most RDEB patients additionally develop carcinomas [43]. A reported prospective phase I/II study that evaluated repetitive intravenous applications of allogeneic MSCs in RDEB children suggests safety of treatment as the primary objective. Only mild adverse events were observed, but none of these led to discontinuation of treatment. Secondary outcome data from skin biopsies do not argue for donor cell chimerism, tissue integration of MSCs, increase in C7 or new anchoring fibrils. Nevertheless, clinical benefits like better wound healing together with reduced skin redness lasting for 4–6 months were reported (EudraCT Nr. 2012-001394-87) [44]. Although low patient numbers (10 individuals included) and a potential for positive information bias due to the unblinded trial design must be taken into account, these encouraging clinical results might have been caused by immunologically active MSC-EVs. Infusion of MSC-exosomes derived from spontaneously differentiated human embryonic stem cells induced M2 phenotype in monocytes in vitro and regulatory T cell polarization in vivo, as well as survival of allogeneic skin grafts in a mouse model [13].

Activation of WNT 4 signaling after application of human umbilical cord (hUC)-MSC-EVs is another possible MoA leading to accelerated skin repair after deep second-degree burn injury in rats, which could be abrogated in vivo by WNT4 knock down [45]. Interestingly, hUC-MSC-EVs seem to promote self-regulation of the WNT/β-catenin signal and may function as accelerators of damaged tissue repair as well as decelerators of WNT signaling via oncoprotein modulation to orchestrate controlled cutaneous regeneration [46]. Systemic application of human adipose tissue-derived MSC-EVs resulted in the recruitment of EV-bearing fibroblasts to the wound areas, increased collagen I and III production and accelerated wound healing in the early stage, followed by reduced collagen expression and reduced scar formation in the late stage of wound healing in a mouse skin incision model [47]. These fundamental findings encourage further clinical testing of MSC-EVs in RDEB patients with local or systemic EV application and the objective to improve wound healing.

### 2.4. Spinal Cord Injury (SCI)

Therapeutic strategies for spinal cord injury, especially after contusion injuries, can either focus on the regeneration of disconnected axons (neuroregeneration) or on the maintenance of the continuity of damaged axons (neuroprotection). Neuroprotective strategies could utilize the fact that injured (but not dissected) axons can persist in a meta-stable state for several hours [48]. Whether or not inherent self-preservation processes can be augmented by local application of allogeneic MSC-EVs in axons, thus using a window of opportunity of about 1–4 hours for rescuing connectivity after non-transsecting SCI, will be of great interest. MicroRNA (miRNA)-133 was downregulated in rat brain after ischemia, and the role of miRNA-133 in mediating SCI repair has been investigated in a zebrafish model [49]. In rat stroke models, beneficial effects for neurite outgrowth could be either transferred by MSCs or their EVs via miRNA-133 shuttle to astrocytes and neurons. Prevention of glial scar formation was induced through miRNA-133-mediated downregulation of connective tissue growth factor [50]. Furthermore, the central role of the exosome-enriched fraction in miRNA-133 delivery, neuronal plasticity and functional recovery after stroke has been confirmed in a rat model [51].

Conflicting in vivo results exist, however, regarding potential pro- or anti-inflammatory events after central nervous system and SCI trauma using murine models. Intralesional injection of MSCs was observed to induce inflammatory activation and to convey beneficial effects in mouse experiments [52]. Similarly, the systemic use of human MSCs or their EVs was equally efficient at preventing post-ischemic immunosuppression, inducing neuroregeneration and promoting successful brain remodeling and functional recovery in a rat model [53]. In contrast, exosomes from rat embryonic cortical neuronal cultures were successfully tested for siRNA delivery to block detrimental effects of inflammasome activation in rats after SCI [54]. Systemic bone marrow (BM)-MSC transplantation was reported to reduce inflammation in the spinal cord by weakening TLR4-mediated signaling and reducing tissue levels of IL-1ß and TNF-α [55]. Rats that were injected with the secretome of hUC mesenchymal progenitors in the hippocampal neurogenic niche displayed similar levels of neural and glial proliferation and differentiation as those injected with parental cells. Moreover, the numbers of doublecortin positive neuronal progenitors in vivo either after MSC or MSC-EV injection were equally increased [56]. Finally, in a rat model of Parkinson’s disease, the secretome of BM-MSCs increased neurogenesis and cell survival and impacted on brain structure and animal behavior [57]. These encouraging data support continued efforts in testing MSC-EVs to alleviate SCI-related symptoms.

If the therapeutic effect after organ damage is expected to support a strong structural regeneration by proliferation of transplanted cells, a potential disadvantage of vesicle-based approaches in comparison to MSC therapy could be the lack of viable cells. The potential benefits of vesicle-based therapies over (stem)cell-based approaches, at least for the above disease indications, reside in the ready availability and ease of storage and distribution of the allogeneic product, the elevated concentration of putative active substances per injectable volume (which is of considerable relevance for early intervention in acute SCI), and the multitude of possible routes and modes of application (such as in wound dressings for EB or in combination with biocompatible natural or synthetic scaffolds for bone regeneration).

## 3. Manufacturing

Internationally harmonized guidelines are available and cover the quality, safety, efficacy and multidisciplinary issues required for drug manufacturing and development. The International Council for Harmonisation of Technical Requirements for Pharmaceuticals for Human Use (ICH) attempts to connect regulatory authorities and the pharmaceutical industry in order to discuss scientific and technical aspects of drug registration at the global level. ICH’s mission is to achieve worldwide harmonization to ensure that safe, effective, and high-quality medicines can be developed and registered efficiently. In this article, we refer to these harmonized ICH guidelines, which are grouped, coded and available online in a logical, consistent and clear way (available online: http://www.ich.org/products/guidelines).

Several essential points should be considered early in the development and setting-up of standard operating procedures for manufacturing, characterization, storage and distribution of EV-based therapeutics (see also Table 2 for MSC-EVs):

(1) At the conception stages of manufacturing and characterization of drugs and for each distinct disease condition, it has to be decided whether a therapeutic is being developed for a small patient population (e.g., rare diseases) or for a large number of potential patients (broad market versus orphan indications). This decision has important implications for the amounts of EVs or VSFs that have to be manufactured and for the general question of scalability, but also for the design and amount of non-clinical (in vitro and in vivo animal) data and clinical testing of the future biological drug.
(2) Large-scale manufacturing has to be planned and evaluated during process and product development to achieve realistic batch sizes for therapy in a clinical setting.
(3) The therapeutic product and its use for treatment can be designed primarily either to address a clear unmet medical need or to compete against multiple existing treatment options.
(4) Depending on the target disease, the route of application (local or systemic use; sole injection of EVs and VSFs; or in combination with cells, medical devices or scaffolds) should be defined early in development.
(5) If human material is used to generate an EV-based therapeutic, arguments favoring either allogeneic or autologous use have to be evaluated in a risk-based approach. The situation may vary with the indications and manufacturing conditions and influence the decision as to whether an allogeneic (broad use, need for instant off-the-shelf availability, relative ease of large-scale production, etc.) or autologous (expected immunogenicity, allergic or toxic reactions, other severe side effects, etc.) strategy is preferred.
(6) The generation of master and/or working cell banks to provide a stable pool of producer cells for EVs can be achieved with media and supplements containing either xenogeneic, human or chemically defined material. The suitability of any reagent for supporting a GMP-compliant process has to be evaluated and confirmed. Safety considerations will favor the use of human material such as pooled human platelet lysate, whereas scalability issues argue for chemically defined media.

### 3.1. Extracellular Vesicles (EVs) or Vesicular Secretome Fractions (VSFs)?

A critical element in the development of future therapeutics is that of regulatory compliance, obtaining a manufacturing license and, finally, a market authorization. For this, product specifications related to “purity, identity, quantity, potency and sterility” need to be defined in accordance with the regulations for pharmaceutical manufacturing. In order to be released for distribution and clinical use, each product batch has to meet predefined quality criteria. If release criteria are not reached because of “out of specification” results indicating deviations from defined and expected quality, the product has to be rejected, and distribution or clinical use of this special batch are prohibited. The issue of quality can be resolved in several ways; and as long as the procedures are validated and the specified ranges match the manufacturer’s requirements, there are few obstacles remaining.

Whatever potency assay is used, the outcome will refer back to product quantification and thus the two issues “quantity and potency” are interrelated. “Sterility” testing follows established procedures for the detection of microbiological contamination and endotoxin levels, and can be enumerated according to validated tests and acceptable threshold levels.

However, when it comes down to “purity and identity”, the issues are less straightforward. The more stringent the definitions for EV-based therapeutics are laid down, the more emphasis is put on purity and identity of the preparation. EVs include a broad variety of membrane-bounded vesicles, while exosomes are restricted, at least, by size and surface markers. EVs either from tissue or from in vitro expanded cell cultures are part of the secretome, which includes soluble molecules like proteins and lipids, extracellular RNA species, and membrane vesicles. Electron microscopy (EM) data show that even highly-purified EV preparations for analytical purposes contain co-purifying components [58]. Considering that large-scale clinical manufacturing of EVs will be less stringent on segregating EVs from co-purifying components, we must expect an increasing fraction of secretome components in the final preparation. In this light the question arises if the definition of EV or exosome is still appropriate, and also how the various secreted components may influence therapeutic activity. If the product is termed “exosomes”, regulatory authorities will require a demonstration of purity, and the percentage of exosomes present in the final product may be in question. In the best case, the product will relate more to an “exosome-containing” preparation.

Such considerations can be discussed at great length, may increase the uncertainty about the vesicle-based therapeutics, and may eventually discourage scientists, manufacturers and potential investors from engaging in clinical trials. But how can this conundrum be resolved? One approach may be to accept the heterogeneity of secretome-based preparations and to find a terminology that embraces all biological components and therapeutic aspects without eliminating the central claim. Secretomes [59] are crude and uncharacterized mixtures of soluble and vesicular components, and yet secretome-based therapeutics are already under pre-clinical investigation [60]. Can secretomes be characterized better than EVs? No. But they are more broadly defined, and as such allow considerable flexibility with regard to purity and identity. From a cell biological standpoint, and not limiting the definition to a purely proteomic view, the secretome can be seen as the totality of organic molecules and inorganic elements secreted by biological cells into the extracellular space, either in a soluble or packaged form.

The manufacturing and enrichment process of EVs eliminates a large portion of soluble secreted proteome components, but the co-purifying fraction is difficult to determine. Nevertheless, the manufacturing strategy aims at enriching (not necessarily purifying) membrane-bounded vesicular structures. It may thus seem more appropriate to consider the resulting product to be a vesicular secretome fraction. The term “fraction” has already been used to identify subclasses of products (e.g., albumin fractions based on the original Cohn procedure) [61]. This terminology can also accommodate fractions containing co-purifying serum components in those cases where the manufacturing process requires the use of (vesicle-depleted) serum.

From the perspective of the manufacturer, the advantages of EV/VSF-based therapeutics over cellular counterparts relate to the possibility of filter sterilization of the final product immediately prior to aseptic filling; a considerable flexibility in the choice of storage buffers; and the reduced demands on the freezing and storage conditions, which substantially reduces the overall costs of the manufacturing process. The disadvantage, in comparison to stem cell therapy, of an extended manufacturing and processing period seems to be well compensated for by the above benefits.

### 3.2. On the Importance of Working with Highly Purified EV Populations

Heterogeneity among EVs not only exists with regard to vesicle size (separating apoptotic bodies, microvesicles and exosomes), but also among exosomal subgroups [62]. It can be anticipated by extrapolation of the natural heterogeneity of the producing cells, and supported by detailed analyses that EV preparations comprise different vesicle populations [63,64,65,66]. Functionality and therapeutic value of EVs or VSFs, however, can only be correlated with their composition once the entire range of secreted EV subpopulations is isolated for thorough analysis and comprehensively described. Thus, it remains to be established how heterogeneity affects the therapeutic effect of a putative biological EV- or VSF-based product [66].

For various investigations, including the definition of a MoA and product release criteria, but also for toxicity, biodistribution or pharmacokinetic studies, highly purified and homogenous EV fractions seem necessary. The technical challenges associated with purifying EVs to homogeneity are evident. For purely analytical purposes, affinity chromatography solutions may be suitable (shiga or cholera toxin binding [63], or heparin affinity binding [67]) and provide satisfactory results. However, such approaches fall short of complying with current GMP requirements and the demand for large-scale enrichment and purification procedures of clinical doses of EVs or VSFs. It appears necessary to define an acceptable purity and identity of EV or VSF preparations with a view to available and manageable purification schemes, and based on serial filtration, tangential flow filtration (TFF) or polymer precipitation. Useful information on the current purification regimens for EVs has been provided, recently [66]. In any case, the overarching perspective must be guided by functionality and clinical efficacy of the therapeutic product, which sets the issue of purity to a second-level of relevance.

### 3.3. Extended Characterization of EVs

Similar to the situation for MSC characterization, a list of present and absent membrane surface markers appears largely insufficient to describe and characterize functional EVs (see Table 1 in [68] and [69]). Rather, a combination of complementary characteristics should be evaluated. In case of MSC-EVs we propose that in addition to the bespoke surface marker profiles, a cytokine profile should accompany EV and VSF characterization in which the potential factors driving immunomodulation, angiogenesis or other intended biologic activities (such as enhanced survival, proliferation or differentiation) can be identified. Finally, a messenger RNA/miRNA profile can point towards a proposed MoA by indicating at least a probability for the regulation of specific pathways leading to the desired therapeutic stimulus.

### 3.4. How to Purify

At present, there is no consensus on the most suitable method for EV enrichment and purification [70]. Major factors influencing the choice of methodology are the starting volume and the intended use of the final product. With the focus of this article on therapeutic application, the logics underlying the choice of enrichment or purification strategy differ from that of analytical and diagnostic use. EV and VSF enrichment from cell culture supernatants (plastic adherent cultures, static or dynamic bioreactors or hollow fiber perfusion reactors) requires an initial volume reduction from the scale of several liters to a few milliliters. For pharmaceutical manufacturing, raw materials or consumables must comply with GMP regulations. Ideally the process is scalable to accommodate future large-scale manufacturing to eventually reduce the costs of goods and to circumvent the risk of unexpected hurdles brought about by procedural changes in late stages of product development. Bearing these parameters in mind, the initial purification steps that follow the clearance of cell debris by low-speed centrifugation and 0.22-µm filtration should include high-throughput filtration steps such as TFF. Standardized GMP-compliant TFF systems are available on the market, offering the possibility of validated process control and documentation.

The TFF process can be used to reduce an initial volume of 10 L to about 200 mL in less than 2 h, depending on the harvest medium used and the molecular weight cutoff chosen. A 750 kDa pore (equaling a 13–15 nm pore size) will eliminate a large portion of soluble, not aggregated, proteins. A buffer change by diafiltration using the same column is advised at this stage. Further volume reduction can be achieved by either additional filtration on smaller sized columns with reduced dead volume, or by low-pressure track etch membrane filtration [71].

A final step employing size exclusion chromatography (SEC) would at this point be suitable for removing protein aggregates and lipoprotein particles. This step, however, commonly causes a significant drop in the total particle number (by 30–70%), owing to the limited recovery, and comes at the expense of a volume increase by a factor of 1.5 [72]. Although upscaling is possible for SEC, this method is currently not suitable for the initial volume reduction required for cell culture-derived EVs and VSFs.

A scalable anion exchange chromatography method yields functionally active EVs [68]. A drawback of this technique, however, is the fact that most serum proteins also bind to the resin and co-elute at 500 mM NaCl with the vesicular fraction. The resulting solution thus equals a vesicle-enriched secretome fraction. Heparin affinity columns [67] seem to more selectively associate with membrane components of EVs, but elution at 2 M NaCl and retrieval of functional EVs appears questionable, and the entire process is time consuming in its present form.

Dynamic culture conditions based on large-scale computer-controlled stirred suspension bioreactors have been applied in an attempt to develop future strategies to manage neurodegeneration involving the use of human MSC secretomes. Secretomes from dynamically cultured BM-MSCs induced a higher number of human neural progenitors to differentiate into neurons compared to MSC secretomes collected under static conditions, and increased the secretion of several neuroregulatory molecules and miRNAs. BM-MSC dynamic secretome further induced neurogenesis, as well as a robust increase in neuronal cell differentiation. These outcomes were associated either with the exclusive presence, or increased expression, of neuroregulatory molecules and miRNAs within the dynamic secretome [56,57].

### 3.5. How is Identity and Purity Defined in EV Preparations?

Irrespective of the technology that is applied for the measurement of EVs, there are pertinent problems to this mode of characterization. Nanoparticle tracking analysis (NTA) and other optical flow-based approaches may quantify the particulate fraction in a solution to a satisfactory level, but are unable to discriminate between particulate and vesicular (membrane-bounded) events. Electron microscopy can solve that problem, but this method is not suitable for quantitative and high-throughput analysis. NTA and flow cytometry techniques combine fluorescent labeling primarily of membrane lipids or transmembrane protein components with enumerable detection of total events. Preliminary developments are promising, but at present do not satisfactorily address the need for a reliable, stable, reproducible and GMP-compliant technology.

A commonly accepted mode of concentration determination is that of total protein content. This approach can only be applied with confidence once the purification process and producer cell lines are standardized and validated. Determining the identity of MSCs is essentially based on the minimal criteria of the International Society for Cellular Therapy (ISCT) set forward in 2006 [73]. The ISCT criteria are still the basic reference for the majority of clinical trials with MSCs and even these rather common surface marker profiles are sometimes diluted out.

If MSCs are measured by flow cytometry and their characteristics are evaluated according to the above ISCT criteria, the majority of MSC preparations reveal an almost 100% MSC identity, as do some fibroblasts (see Table 2 in [74]). However, by adding only 1 or 2 other surface markers, this seemingly homogeneous population reveals a striking heterogeneity [75,76].

As a consequence, the majority of MSC populations should be regarded as non-clonal and heterogeneous with unpredictable properties [77] and an average composition of at least three different cell populations. If such cell pools secrete only two functionally different EV populations (e.g., exosomes and microvesicles), which may further comprise two or three different exosome fractions, the identity of one particular EV fraction in a seemingly homogeneous EV pool drops below 10%. These considerations once more emphasize the need to reconsider the applied terminology and to perhaps use the term VSF instead of EV. As long as identity and purity of a therapeutically active drug cannot be better defined, a common principle for early development of biologicals predicts that “*the process is the product”*. We suggest that this consideration is also valid for EV- or VSF-based therapeutics.

### 3.6. Release Criteria

If indeed the process is the product, then the release criteria for MSC-EVs must encompass both the producing cells and the enrichment scheme. Minimal acceptance and release criteria may thus include the following:

(1) MSCs display an ISCT-compliant surface marker profile at the time of secretome harvest [73].
(2) EVs within the secretome fraction must comply with the minimal criteria of ISEV, at least for a number of membrane markers [78].
(3) The size range should be in the range of exosomes (50–150 nm).
(4) Sterility and endotoxin levels must comply with regulatory requirements.

For a stable and compliant process, protein concentration as a means to determine the concentration of given EV preparation may not be a suitable release criterion. Co-purifying proteins from the secretome or serum components prevent a precise analysis of the EV-associated protein moiety. Moreover, if an anticipated therapeutic activity and EV quantification are based on, for example, MHC class II molecules [3], the question may arise as to why the preparation is not defined as an MHC II product containing lipids and RNA, instead of a vesicle-containing product. While the protein profiles of MSCs and their EVs or VSFs remain astonishingly constant despite largely divergent culture conditions and enrichment procedures, the miRNA content changes within a few h when culture conditions are altered. Thus, a basic profile for miRNA should be established for each method of cell cultivation to accommodate all preferences (serum type, medium type, oxygen levels, etc.). The reproducibility of miRNA profiles may be best suited to report on the persistence of a stable manufacturing procedure (irrespective of a complete lack of prediction for a MoA by the miRNAs).

### 3.7. Naive vs Loaded EVs

An alternative approach to using unmanipulated MSC-EVs is that of loading vesicles with a known and characterized active component (proteins or nucleic acid-based compounds). However, similar regulatory requirements with regard to robustness, reproducibility and scalability of the loading and manufacturing process also apply for loaded EVs [79] and the loading methods. A series of siRNA loading technologies have been tested including transfection, electroporation (can lead to RNA aggregation), co-incubation of cholesterol-conjugated siRNA [79], and liposome fusion with hybrid biocompatible carriers (Anjarium’s proprietary Hybridosome™ technology) that comprise structural and bioactive elements and a tunable fusogenic moiety. DNA loading via electroporation seems to be the current standard, resulting in the presence of several hundred linear DNA molecules (less than 1000 bp) per EV [15]. Vesicle loading with chemotherapeutics by electroporation, incubation and mild sonication has gained increasing popularity, and encouraging results for Paclitaxel loading demonstrate that exoPTX performs better than Taxol, as judged by the antineoplastic efficacy in a Lewis Lung Carcinoma mouse model [80].

Irrespective of these seemingly direct therapeutic approaches, ex vivo loaded EVs also have to be prepared from source cells and cannot (at present) be synthesized from artificial components. The most popular cell lines currently employed are RAW 264.7 macrophages, various dendritic cells, glioblastoma and lung carcinoma lines, and HEK 293 cells. It appears necessary to point out that all co-purifying components in enriched vesicle fractions from these cell sources may contribute to the overall therapeutic effect in addition to that of the loaded substance.

It has to be considered that tumor cells are able to transform not only neighboring cells by transferring proteins and nucleic acids, but also the extracellular matrix by releasing several metalloproteases, potentially via EVs. Therefore, the use of tumor cells in the manufacturing of EVs or VSFs for therapy may convey pro-tumorigenic effects that have to be investigated with sufficient caution prior to clinical evaluation. Furthermore, any side effects and immunological reactions may be also caused by the vesicular drug delivery systems and effects of such combined therapeutic products must be well characterized prior to application for market authorization.

### 3.8. Aseptic Filling, Storage and Stability of the Final Product

Aseptic filling in non-automated manufacturing sites requires an A-in-B cleanroom environment (class 100 in class 1000 by U.S. standards). Potential manufacturers should bear this in mind during the planning phase. The biophysical properties of EVs and VSFs require low adsorption materials to be used as storage containers. Depending on the formulation of the final product, such packaging material must be validated, and the suitability confirmed. Currently, some low-protein binding synthetic materials are available from several suppliers, but recommendations supported by conclusive data from manufacturers of EV therapeutics are still missing.

If EVs are stored as highly-concentrated, ready-to-use liquid formulations, the storage temperature has to be determined in order to preserve the monodisperse suspension and to avoid aggregation or degradation over time. While in most cases a temperature between −40 and −80 °C seems appropriate, the lower temperature range raises concerns regarding the ease of shipment and distribution of the product, as well as available storage options at the points of care. Thus, stability testing programs need to be executed that address not only the long-term storage conditions but also the potential influence of changing temperatures during storage and distribution until the administration of the product to patients. For all the above issues we recommend a thorough and well-documented risk-based analysis to comply with practical issues of clinical application and regulatory requirements.

### 3.9. Biodistribution, Bioavailability, Cytotoxicity and Pharmacokinetics

Administration of EVs in patients has already been tested in a small number of phase I clinical trials, revealing an overall low toxicity and considerable stability of EVs in the circulation [4,5,6,81,82]. Currently, the original research information on the biodistribution of EVs is extraordinarily scarce [83,84,85]. The use of clinically approved radioisotopes for labeling EVs seems a fruitful approach. Supramagnetic particle loading is an alternative, but both techniques bear considerable limitations. A significant contribution to this matter was shown recently [86]. Based on this work and that of others [83,87], it appears that EVs are rapidly sequestered (within minutes) by circulating macrophages. Thus, systemic (intravenous) application of EVs may not be the best route for achieving significant therapeutic effects. More solid data from both pre-clinical and early clinical phase I studies are urgently required to inform the community about this important aspect, and to improve future therapeutic applications.

### 3.10. Predictive Potency Assays, Mode of Action (MoA) and Proof-of-Concept (PoC)

A basic biological readout to evaluate the in vitro and in vivo therapeutic potency of EVs and VSFs seems mandatory. However, there is currently no consensus about the biological relevance of EVs. If we focus only on one possible active EV-component, such as RNAs, and on how one may effectively and reliably characterize them in an EV preparation, the current knowledge prevents the implementation of universally acceptable standards for EV-RNA analysis as part of the product release criteria [88], and this seems to be true for the entire EV field. Combinatorial potency assays should be developed to account for the multifactorial regenerative instructive potential that can be exerted by MSC-EVs. As for the few model target diseases that we discuss in this article, it is understood that predictive potency assays for bone regeneration should not only depict the investigational drug’s capacity for inducing osteoblast proliferation, but should also take into account a potentially required osteoclast activity and/or angiogenesis. Potency assays for skin repair in EB have to go beyond a 2D keratinocyte migration assay, and in the case of acute SCI models potency testing for neuronal outgrowth may have to be supplemented with assays depicting the cellular re-myelination propensity, as well as an overall immunomodulatory and neuroprotective capacity.

One more essential question that needs to be addressed is the proposed MoA of EVs or VSFs in any of the intended applications. At least a rudimentary knowledge of a potential MoA is obligatory for designing a suitable test environment for the examination of a novel drug substance in relevant animal models, and to provide meaningful PoC studies [68,69].

However, even if no definitive MoA can be described and the active component of a given novel biological product that is responsible for an observed therapeutic activity remains ill-described, it may be possible to engage in early clinical trials for indications of a definitive unmet medical need. Poorly characterized biological therapeutics in early developmental stages can be clinically tested after thorough safety evaluation and the reproducible and convincing demonstration of a therapeutic effect in vitro and in relevant animal models.

A prerequisite for this strategy, however, is that the investigative product is of reproducible quality and biological effect, irrespective of the precise content and function of the individual factors. Once again, we may have to look at the manufacturing process as the therapeutic product.

Prockop and colleagues recently provided a striking correlation between MSC treatment and the (dose-dependent) presence of the anti-inflammatory protein TSG-6 [76,89]. The group confirmed a proposed anti-inflammatory MoA via TSG-6 that is responsible for the positive therapeutic effect of MSCs after myocardial infarction and chemical cornea injury in mouse models, but this criterion is not yet broadly applied when therapeutically active MSCs are characterized.

Investigating the potency of MSC-derived EVs in post ischemic stroke, a PoC for the potential neuroprotective and neuroregenerative capacity of EVs was provided [53]. EVs successfully recapitulated the effect of MSCs, and no adverse reactions were reported. In a compassionate clinical use experiment to treat GvHD, a similar level of safety and efficacy for MSC-derived EVs was demonstrated. This first-in-man approach suggests a promising future for phase I and II clinical studies to test the systemic application of MSC-EVs [90].

## 4. Academia and Biotech-Companies: A Discovery Partnership

With the ultimate goal of bringing EV-based therapies into the clinic, the route to product development and pharmaceutical production needs to be planned with caution and foresight. Pharmaceutical R&D bears a Pandora’s box of traps and problems, and technology transfer for industrial scale manufacturing is only one of them that we have alluded to above. Among the further stumbling blocks are a common lack of reliability of published data (mostly caused by non-standardized manufacturing processes and inadequate characterization), a complete lack of adequate or poorly predictive pre-clinical animal models and potency assays, competition for proprietary targets, and the inherent complexity of target validation for cell-based biologicals. In clinical research, the roadblocks relate to underestimation and misinterpretation of the complexity of clinical trials, a lack of know-how of smaller, mostly academic, organizations with regard to GMP manufacturing, trial execution combined with wrongly defined study endpoints, and flawed clinical trial reporting and documentation [91]. Regulatory constraints do not constitute unnecessary hurdles, and should be recognized as helpful guidelines for translational considerations during the development and transfer of basic discovery into meaningful clinical trial procedures [92].

Academic-industrial partnerships should also be considered to be fruitful alliances on the road to the clinic with vesicle-based therapeutics. Glaxo-Smith-Kline (GSK) has successfully modified its R&D strategy by collaborating with academia to the benefit of both parties [91]. Through similar approaches large pharmaceutical entities gain access to ideas and innovation, while academia can exploit cost-intensive drug and target discovery, or preclinical safety. Further partnerships with rare disease-focused foundations may moreover allow the industrial and academic partners to gain access to additional funding in a process known as “venture philanthropy”, and make R&D more efficient and affordable [93]. The value of innovation centers for integrating know-how from different aspects of product development and pharmaceutical manufacturing, including legal and regulatory issues, is still underestimated in academia.

## 5. Conclusions

As much as the entire field of regenerative medicine, the development of EV- or VSF-based therapeutics has a huge but mostly unexploited potential. Despite its biological and regulatory complexity, it is mandatory to continue the quest for developing scalable and reproducible purification protocols based on robust risk-based approaches, and to elucidate the MoA through qualified potency assays in disease-relevant in vitro and in vivo models. If GMP compliance and a well-developed understanding of the benefit of regulatory requirements can be applied to EV- or VSF-based biological therapeutics development, we will see a number of valuable clinical trials with EV products in the near future. Regulatory compliance is an integral part of a stable manufacturing process, and if indeed “the process is the product”, this compliance will remain a trustworthy bridge on the crooked way to pharmaceutical production for the benefit of the patients.

## Figures and Tables

**Table 1:** List of companies offering exosome-/extracellular vesicle- or secretome-based services and products.

Company Name	Therapeutic Target	Technology/Product	Url Web Address
Anjarium Biosciences	Broad range of severe diseases	“Hybridosome^TM^” for targeted delivery of drugs	http://anjarium.com/
Aposcience AG	Stroke, spinal cord injury, skin lesions, acute andchronic myocardial infarction	Peripheral blood mononuclear cell secretome “APOSEC^TM^”	http://www.aposcience.at/the-secretome-company/
Capricor Therapeutics	Cardiovascular and non-cardiovascular diseases	Cardiosphere-derived cells “CAP-1002” and exosomes thereof “CAP-2003”	http://capricor.com/
Codiak Biosciences	Pancreatic cancer	Exosomes for targeted drug delivery and diagnostic application; exosome origin not indicated	http://www.codiakbio.com/
Esperite Group/ The Cell Factory	Various diseases from neurology to orthopedics	MSCs and MSC-derived EVs and exosomes	http://www.esperite.com/?page_id=13
			http://www.cell-factory.com/
Evothera	Unclear portfolio	Not indicated	Not found
Evox Therapeutics	Serious life-threatening diseases, first focus on inflammatory and neurological diseases	Loaded exosomes for targeted delivery; exosome origin not indicated	https://www.evoxtherapeutics.com/
ExoCyte Therapeutics	Cancer	Cancer vaccines: Autologous DCs electroporated with tumor-derived exosomes, co-administered with checkpoint inhibitor	http://exocytetherapeutics.com/
Exogenus Therapeutics	Skin lesions	Exosome-based product “Exo-Wound”; exosome origin not indicated	http://www.exogenus-t.com/
Exovita Biosciences	Diverse Cancers	Therapies based on exosomes, which are cytotoxic to cancer cells	http://exovitabio.com/
Kimera Labs	Orthopedic, cosmetic and regenerative medicine applications	MSC-derived exosomes “XoGlo^TM^”, amniotic fluid-derived product “Amnio2x^TM^”	http://kimeralabs.com/
Med Cell Europe *	Orthopedic, neurologic, ophthamologic, and cardiologic diseases, anti-aging application	Adipose tissue-derived stem cells and secretome	http://www.medcelleurope.com/ *
Paracrine Therapeutics	Stroke, myocardial infarction, osteochondral defect, graft-versus-host disease	Embryonic stem cell-derived MCS-EVs	http://paracrinetherapeutics.com/
ReCyte Therapeutics	Vascular disorders	Embryonic progenitor cells and their secreted factors, including EVs	http://www.recyte.com/
ReNeuron	Neurologic and ophthalmologic disorders	Retinal progenitor cells, neural stem cells and EVs thereof	http://www.reneuron.com/
Stemedica Cell Technologies, Inc.	Cardiovascular diseases, traumatic brain injury, cutaneous photoaging, Alzheimer’s disease	Ischemia-tolerant MSCs and neural stem cells; stem cell factors from MSCs	https://www.stemedica.com/
ZenBio	Skin lesions	Exosomes from pre-adipocytes, placental MSCs and cord blood serum	http://www.zen-bio.com/

* According to this web site, Med Cell Europe has discontinued all activities. The probable reason for this is a legal issue (further information are available online: http://www.tagesanzeiger.ch/schweiz/standard/im-clinch-mit-swissmedic/story/14386207 and http://www.derbund.ch/schweiz/standard/so-werden-die-umstrittenen-stammzellen-gespritzt/story/20046763?track).

**Table 2:** Considerations for the manufacture of mesenchymal stromal cell-derived extracellular vesicles (MSC-EVs) for selected therapeutic applications. For any therapeutic application of MSC-EVs, we principally suggest manufacturing under xenogenic substance-free conditions, and under consideration of the pros and cons of allogeneic use.

Disease	Predicted Market Size	Medical Need	Application Route	Amount/Dose
Critical size bone defect	common disease, broad market	unmet	local via scaffolds	large
Epidermolysis bullosa	rare disease, restricted market	unmet	local via wound dressing	small
			systemic	large
Spinal cord injury	rare disease, restricted market	unmet	local without scaffold	small
			systemic	large

## Abbreviations

BM	bone marrow
C7	type 7 collagen
COST	Cooperation in Science and Technology
DCs	dendritic cells
EB	epidermolysis bullosa
EM	electron microscopy
EVs	extracellular vesicles
GMP	good manufacturing practice
hUC	human umbilical cord
ICH	International Council for Harmonisation of Technical Requirements for Pharmaceuticals for Human Use
ISCT	International Society for Cellular Therapy
ISEV	International Society for Extracellular Vesicles
Me-HAD	European Network on Microvesicles and Exosomes in Health and Disease
miRNA	microRNA
MoA	mode of action
MSCs	mesenchymal stromal cells
NTA	nanoparticle tracking analysis
RDEB	recessive dystrophic epidermolysis bullosa
PoC	proof of concept
SCI	spinal cord injury
SEC	size exclusion chromatography
TFF	tangential flow filtration
VSFs	vesicular secretome fractions
